# Low-Dose Radiation Therapy (LDRT) for Lymphatic Obliteration in Persistent Chylous Ascites: A Case Report

**DOI:** 10.7759/cureus.98646

**Published:** 2025-12-07

**Authors:** Eduardo Guerrero Lizcano, Yuli Natalia Otero Pabon, Javier Rodriguez Corredor, Santiago Ardila Giraldo, Andrés González Ramírez, Sofía Dávila Garzón

**Affiliations:** 1 Radiation Oncology, Instituto Nacional de Cancerología, Bogotá, COL; 2 Radiation Oncology, Instituto Médico Oncológico, Neiva, COL; 3 Nuclear Medicine, Instituto Nacional de Cancerología, Bogotá, COL; 4 Internal Medicine, Hospital San Juan de Dios, Armenia, COL

**Keywords:** lymphatic fistula, lymphoscintigraphy, postoperative chylous ascites, radiation theraphy, refractory ascites

## Abstract

Chylous ascites is a rare postoperative complication, typically associated with extensive abdominal procedures and caused by inadvertent injury to the retroperitoneal lymphatic vessels. Its management represents a therapeutic challenge, particularly in cases refractory to conventional strategies. We present the case of a patient with persistent lymphatic leakage following retroperitoneal lymphadenectomy for recurrent testicular cancer, who failed multiple standard interventions. Given the refractory nature of the condition, low-dose external beam radiotherapy was administered, resulting in complete resolution with no recurrence at two-year follow-up. This case highlights radiotherapy as a potentially effective therapeutic option for the management of lymphatic fistulas.

## Introduction

Chylous ascites is an uncommon postoperative complication [1], resulting from inadvertent disruption of the retroperitoneal lymphatic vessel, leading to an abnormal communication between major retroperitoneal lymphatic channels and the peritoneal cavity, causing lymph leakage and accumulation [2]. Its incidence varies depending on the type of surgery: 7% following complex abdominal procedures [3], 9% after retroperitoneal lymphadenectomy [4], 5% following laparoscopic nephrectomy [5], and 1.2% after lymph node dissection for testicular cancer [6]. Due to its rarity, there is no consensus on standardized diagnostic and therapeutic approaches, making its management particularly challenging [2].

We report the case of a patient with a history of testicular cancer who underwent retroperitoneal surgery for tumor recurrence and subsequently developed postoperative chylous ascites refractory to conventional treatment (nutritional suspension, embolization, surgical ligation), which was successfully managed with radiotherapy.

## Case presentation

A 32-year-old male was presented in September 2021, with a diagnosis of mixed germ cell tumor (50% seminoma and 50% mature teratoma) post right radical orchiectomy, stage IS (pT1NxM0S1), with no adjuvant therapy. Five months later, serum lactate dehydrogenase (LDH) levels were found to be elevated, and a computed tomography (CT) scan revealed a 6 cm retroperitoneal mass in the right renal parahilar region with circumferential infiltration of the ipsilateral artery and vein. The patient received three cycles of bleomycin, etoposide, and cisplatin, achieving normalization of tumor markers; however, retroperitoneal disease still persisted. An open retroperitoneal lymphadenectomy was performed, with removal of seven lymph nodes and two lymph node conglomerates; histopathological analysis confirmed pure seminoma involvement in the conglomerates of lymph nodes; the remaining lymph nodes were reported to be tumor-free.

On postoperative day five, the patient presented with mild abdominal distension and chylous fluid drainage from the lower third of the surgical wound, with a progressively increasing volume. Oral intake was suspended. CT imaging revealed moderate ascites. An enterostomal drainage of the fluid was performed, and analysis showed a triglyceride level of 286 mg/dL, confirming postoperative chyloperitoneum secondary to lymphatic fistula. 

Initial treatment included percutaneous embolization of retroperitoneal lymphatic vessels, but due to persistent leakage, further interventions were attempted, including surgical ligation and a second embolization, both unsuccessful. The patient experienced increasing abdominal distension and significant deterioration in quality of life, with daily drainage volumes reaching up to 2600 mL. 

**Figure 1:**
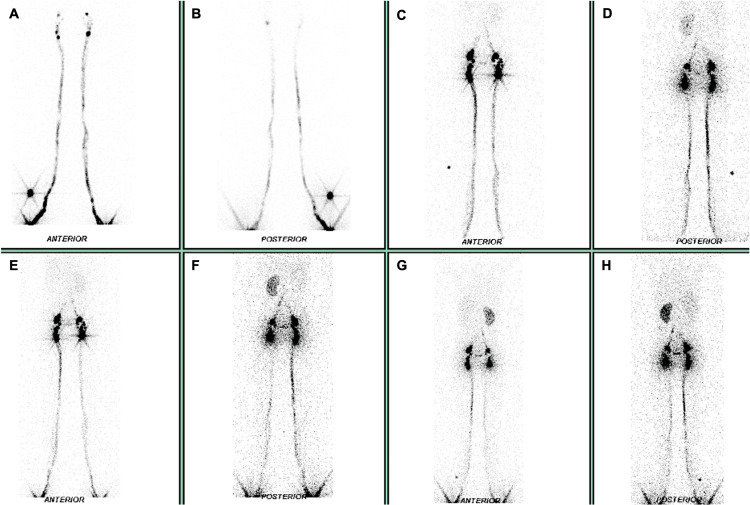
Lymphoscintigraphy A total of 55 MBq of technetium-99m-labeled nanocolloids was administered via intradermal injection into the first interdigital spaces of both feet. Planar imaging was performed using a SIEMENS NM Encore 2 gamma camera in anterior and posterior projections of the lower limbs, pelvis, abdomen, and thorax immediately after injection and at one-, two-, and three-hour post-injection intervals. Images (A) and (B) show the normal ascent of the radiotracer up to the pelvic region. In (C), radiotracer accumulation is observed in inguinal and pelvic lymph nodes with a physiological appearance. In (D) and (E), physiological visualization of both kidneys is noted. In (F), (G), and (H), there is an absence of proximal radiotracer passage at the L4 level. Hepatic visualization was poor, likely due to the lack of proximal migration of the radiotracer beyond the aforementioned level.

**Figure 2:**
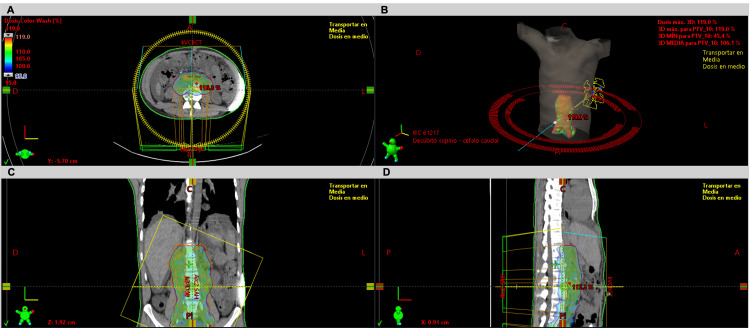
External beam radiation therapy Simulation CT scan performed with the patient in the supine position, arms raised above the head and immobilized using a Vac-Lok system to ensure treatment reproducibility. No gross tumor volume (GTV) was defined. The clinical target volume (CTV) included retroperitoneal lymphatic drainage along and around the aorta and inferior vena cava, extending from the T12 to the L5 vertebral levels. The planning target volume (PTV) was generated by adding a 5-mm margin to the CTV. The dosimetric plan was generated in the Eclipse™ Treatment Planning System v16 (Varian Medical Systems) using the Acuros XB algorithm. In the axial (A), coronal (C), and sagittal (D) views, 95% of the prescribed dose coverage over the PTV is observed. The maximum dose to the spinal canal was 10.65 Gy, while the spinal cord received 7.62 Gy. The mean kidney doses were 4.38 Gy (left) and 5.69 Gy (right), remaining within tolerance limits. A volumetric modulated arc therapy (VMAT) technique with two arcs (B) was used as a treatment technique.

Given the refractory nature of the condition despite multiple conservative interventions, an extensive literature review was conducted, and the multidisciplinary team decided to proceed with external beam radiotherapy as an alternative therapeutic approach. Prior to treatment initiation, lymphoscintigraphy was performed to localize the fistula, revealing absent radiotracer migration in the left para-aortic retroperitoneal region at the L4 level (Figure 1). Based on these findings, a volumetric modulated arc therapy (VMAT) plan was developed, prescribing a total dose of 10 Gy delivered in 10 daily fractions of 1 Gy, five days per week. The treatment volume included the retroperitoneal lymphatic drainage along and around the aorta and inferior vena cava, extending from vertebral levels T12 to L5 (Figure 2). The patient completed treatment without complications. Given the time elapsed since surgery and the postoperative complication, no chemotherapy was administered.

**Figure 3:**
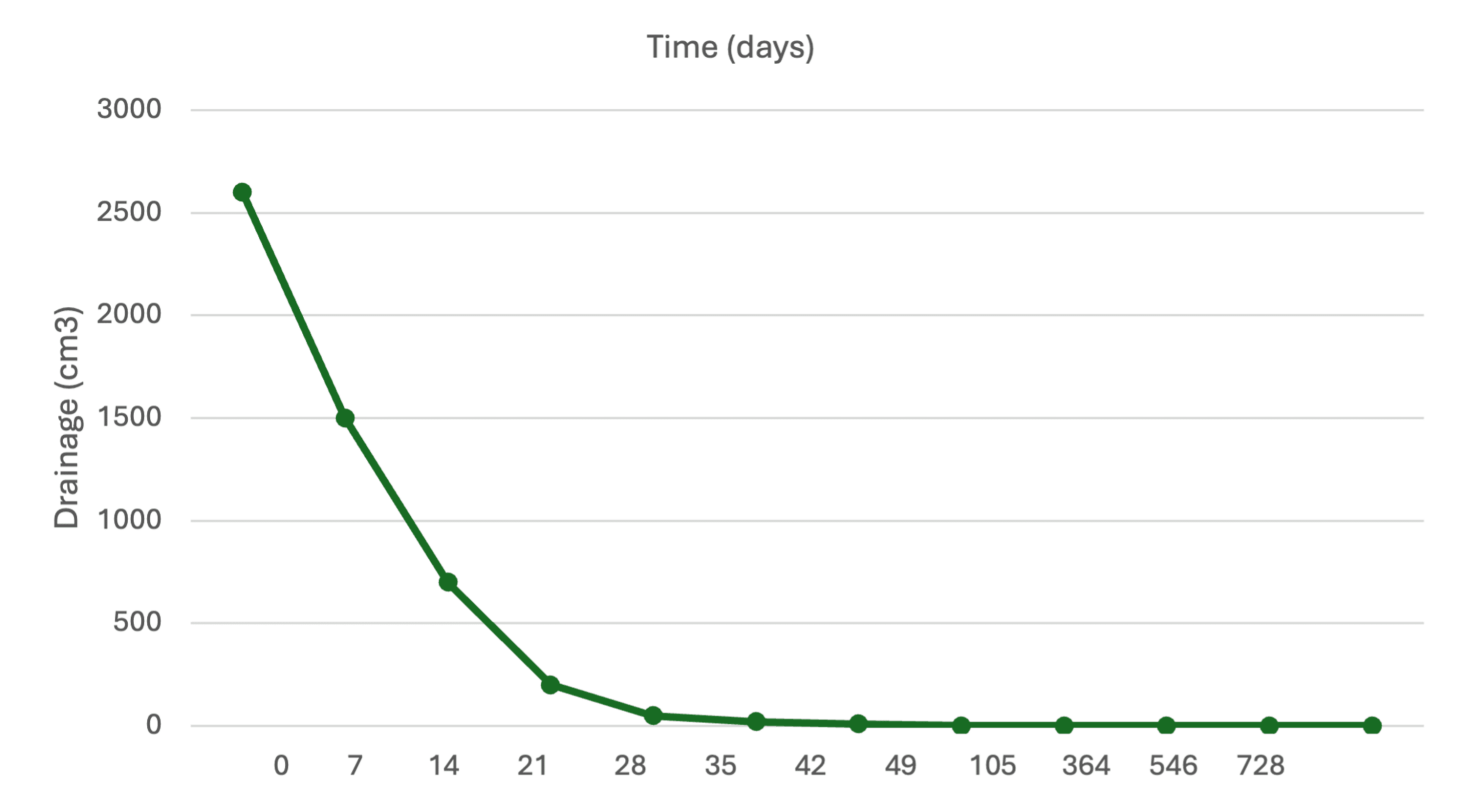
Evolution of drainage after radiotherapy treatment

At 24 months after completing radiotherapy, the patient remains free of fluid drainage (Figure 3), with no measurable disease, negative tumor markers, and an adequate quality of life. 

## Discussion

Postoperative chylous ascites is a rare but challenging complication associated with increased morbidity, higher risk of infections, and prolonged hospital stays [7]. In addition to surgical causes, chylous ascites may result from congenital lymphatic abnormalities (more common in the pediatric population), peritoneal infections, liver cirrhosis, or malignant neoplasms [8]. When surgery is the cause, the injury typically involves the thoracic duct, the cisterna chyli, or their main tributaries, although the overall incidence remains low [9]. 

Diagnosis is based on a combination of clinical findings and ascitic fluid analysis. Symptoms may include peritonitis and ileus, while abdominal distension is less common [3]. Triglyceride levels two to eight times higher than plasma, along with lymphocyte-predominant cellularity, are characteristic of chyloperitoneum [2]. Imaging may help localize the site of lymphatic leakage. Although CT has low specificity for chylous ascites, rupture of the cisterna chyli may be suggested by the presence of retroperitoneal or peritoneal collections [10]. Lymphangiography is recommended in unclear cases, while lymphoscintigraphy with Technetium-99m is considered a less invasive and safer alternative for evaluating abnormal lymphatic drainage [11]. 

Delayed diagnosis and treatment can lead to complications such as dehydration, weight loss, and even sepsis [3,7]. In oncologic patients, these complications represent an additional challenge, as they may delay the administration of adjuvant therapy [1]. 

Initial management is based on conservative measures, including dietary restriction, somatostatin administration, and peritoneal drainage, aiming to reduce lymphatic leakage and preserve the patient's nutritional status. In refractory cases, lymphangiography with embolization has been described as an effective therapeutic option, though not without risk of complications. Surgery is typically reserved as a last resort [1]. 

For patients with persistent fistulas, low-dose radiotherapy (LDRT) has emerged as an effective therapeutic alternative for obliterating the lymphatic tract [12]. Its radiobiological effect has been attributed mainly to two mechanisms: anti-inflammatory and antiproliferative [13]. Doses between 2 and 6 Gy exert an anti-inflammatory effect by inhibiting inducible nitric oxide synthase (iNOS) expression, reducing endothelial cell-leukocyte interactions, decreasing vasodilation, and promoting the release of anti-inflammatory cytokines such as IL-10 and TGF-β1 [14]. The antiproliferative mechanism occurs at doses between 8 and 10 Gy, which induce cell cycle arrest, limiting proliferation in the irradiated tissues. At doses exceeding 10 Gy, an additional immunomodulatory effect has been described, involving the regulation of lymphocyte antigenic stimulation and suppression of local autoimmune processes [13]. Furthermore, it has been proposed that low-dose radiation may also exert functional modifications at the vascular level, such as downregulation of E-selectin in endothelial cells, diminished leukocyte adhesion, and reduced L-selectin expression, contributing to its anti-inflammatory action [7]. 

There is no established standard radiotherapy dose for the management of lymphatic fistulas. However, the German Society for Radiation Oncology (DEGRO) S2e guidelines for radiotherapy in benign diseases (version 3.0) recommend low single doses of 0.3-0.5 Gy, as available data suggest better responses with these doses [15]. Mayer et al. [7] reported the use of radiotherapy in 17 patients, resulting in successful closure in 15 cases with fractionation schemes of 0.3-0.5 Gy per session up to a total dose of 10-12 Gy. Similarly, Neu et al. [16] reported a 96% success rate (27 of 28 patients) with total doses ranging from 3 to 12 Gy. Kim et al. [17] described a case of chylous ascites after total gastrectomy in which 8 Gy was delivered in eight fractions, attaining favorable control of the leak after conservative treatment had failed. Likewise, Dilek et al. [18] documented complete resolution of a refractory chylous fistula secondary to hepatectomy with 1.5 Gy per session to a total dose of 12 Gy, with complete response observed 28 days after treatment. In our case, the patient received 10 Gy in daily 1-Gy fractions, achieving complete resolution of chyloperitoneum 40 days after treatment and remaining recurrence-free during two years of follow-up. These doses are consistent with those of Torres et al. [13], who recommend a dose per fraction of 0.5-1 Gy administered four to five times per week, up to total doses of 3-10 Gy, for the management of lymphatic fistulas.

The limitations of this study are related to its case-report nature, which prevents generalization of the findings and limits the availability of comparative data due to the absence of a control group. Therefore, it is not possible to definitively conclude that LDRT is superior to other treatments that have failed to elicit a response.

## Conclusions

Postoperative chylous ascites is an uncommon complication of retroperitoneal surgery associated with high morbidity and represents a therapeutic challenge due to the absence of standardized management protocols. In the present case, LDRT achieved complete resolution of chyloperitoneum after the failure of conservative measures (oral suspension, twice embolization and surgical ligation), with an excellent clinical outcome and no recurrence during follow-up.

Although current evidence remains limited, LDRT appears to be a proven salvage therapeutic option in refractory cases. Further studies are warranted to optimize dose and fractionation regimens and to establish selection criteria for the systematic incorporation of radiotherapy into the management of lymphatic fistulas.
